# Notes and News

**Published:** 1890-10-25

**Authors:** 


### Notes and News.

Collected and Communicated.

Some epigrammatist has advanced the theory that the unhousing of the working classes is a more serious question than the housing of the working classes. Doubtless Irish members will agree. But without entering into the woeful tale of the evictions going on in the Emerald Isle, is there not a grain of truth in the theory so far as it concerns London? We "unhouse" our working man who is living in an insanitary dwelling and leave him to go—we care not where! The Fabian Society bids us run free trains for working-men; who knows but the County Council may in time adopt that plan, and bring into London every morning great batches of navvies who have spent the night in some rural village. This is an age of progress, and we ought not to be content to "unhouse" our working man and then leave him to shift for himself.

A rather wide-prevailing discontent amongst lunatic asylum officers has found expression at Stockport, for at the late meeting of the Board of Guardians a letter was read from Dr. John Brown, of Burwood House, Bacup, stating that he had read in the Manchester *Examiner and Times*, the remarks made last week at the Board's meeting by Councillor Morley, in reference to the paucity of medical attendants at the County Lunatic Asylum at Whittingham, near Preston. He agreed with Mr. Morley that there should be a larger proportion than three doctors to 1,810 patients, and he took this opportunity of giving Mr. Morley every support in his protest. The *British Medical Journal* last year called attention to the fact that the curative treatment was a greater success in asylums in Austria than in England. He (Dr. Brown) attributed this to the insufficiency of medical men at many of our asylums, careful curative treatment therefore not being possible. Mr. Morley said the matter was a very proper one for the guardians to discuss. The medical superintendent (Dr. Wallis), at Whittingham, had told him that if the medical staff was larger more patients would recover. With but one doctor for every 600 patients it was, urged Dr. Wallis, impossible to give each case that attention called for. It was agreed that Dr. Brown's letter be acknowledged, but no further action was taken. Boards of guardians are stolid as a rule; it requires a deal of hammering to produce any impression on them.

The New South Wales papers give a brilliant account of the opening of the Carrington Convalescent Hospital at Camden, near Sydney. The ceremony was performed by Lady Carrington on August 20th, and there was a very large gathering of fashionable people, for the weather was all that could be desired. Sir Alfred Roberts, standing on the porch, asked Lady Carrington to open the building, which thereafter he hoped would recuperate the strength of many bread-winners and bring happiness to many homes. Sydney had long felt the need of a hospital home, and the idea of its foundation originated with Mr. W. H. Paling, who, in a letter to the Governor, dated January 17th, 1888, generously offered to him, on behalf of the colony, the valuable Grasmere Estate of 560 acres, with its lake of 7 acres, stock, buildings, and improvements, together with the noble sum of £10,000. The hospital had since been completed, and thus a temporary home had been provided for those reduced by severe illness, who would be selected without regard to creed or nationality. The building had been skilfully designed by the architect (Mr. Kent). Lady Carrington opened the door with a gold key, and inspected the whole building. The matron then gave a hearty welcome to a batch of convalescents, who arrived by special train.

The Newport Town Council have been holding a delightful discussion on the Diseases Notification Act; we regret we cannot amuse our readers by giving it in full, but the following samples will suffice to show what knowledge and common-sense were brought to bear on the subject. Mr. ——(no, we will so far spare him, we will not print his name). Mr. —— said, in reference to a scarlet-fever case, "Dr. Groves would no doubt have consigned that girl to an infectious hospital in London, and God only knows what would have happened to her there. She would have been dead and buried by this time." The next speaker was even more enlightened; he said that one great objection he had to the Act was its interference with private life, carrying away patients from their homes to hospitals, where they would be placed at the mercy of trained nurses, or of more ignorant persons to whom the work was delegated. Hear, oh ye nurses! for such is fame; you are regarded as one step worse than the old ignorant holders of your title. One part of the discussion with reference to the Ganges, is too absurd to quote, for to accuse the Notification Act of being in any way similar to the killing off of the aged in India, is stupidity too dense to be amusing. The adoption of the Act was negatived by nine votes to eight. We send our sincere sympathy to Dr. Coombs.

Sir Morell Mackenzie has been to the Crimea on the *Chimborazo*, and while he was enjoying a most delightful voyage, appeared his sensational article in the *Contemporary* on hospital dangers. Speaking of our larger hospitals, he says: "In the first place, they are an unscientific anachronism, the crowding together of such a vast number of diseased persons being as much out of place in cities as intramural burial of the dead. Indeed, it is extremely likely that the germs derived from such accumulations of every form of disease are more dangerous to the community than those which, after several years, may emanate from dead bodies. Sir James Simpson, many years ago, called attention to the dangers of what he called "hospitalism"—that is to say, the peculiar unhealthiness caused by large aggregations of persons suffering from different kinds of diseases, who poison the air with their exhalations; and there is no doubt that in many cases they exchange microbes till recovery often becomes difficult for even the strongest." The truth at the root of these words is known to every medical man, but you ask any practitioner whether he would rather perform an operation on a poor patient in the home or the hospital. Danger is comparative, Sir Morell Mackenzie; if hospitals are dangerous for the sick, still more so are unsanitary homes.

The following vacancies are announced this week, the amount of the salary in each case being given after the name of the institution:—

**Resident Medical Officers.**

Paddington Green Children's Hospital, House Surgeon—£50, board, &c.
Great Northern Central Hospital, House Physician—£60, board, &c.
Sheffield Public Hospital, Junior Assistant House Surgeon—£50, board, &c.
Norfolk and Norwich Hospital, Assistant House Surgeon—board, &c.
Clayton Hospital, Junior House Surgeon—£25, board, &c.
Bradford Infirmary, House Physician—£100, board, &c.
Liverpool Dispensaries, Head Surgeon—£200, rooms, etc.
Brentwood Asylum, Junior Assistant Medical Officer—£100, board, &c.

**Non-Resident Medical Officers.**

Loughboro' Medical Aid Association, Junior Medical Officer—£160.
Walsingham Union, Medical Officer—£38.
Ancoats Hospital, Manchester, Honorary Physician.
National Hospital, Queen Square, Registrar and Pathologist—£50.
City of London Hospital, Victoria Park, Pathologist—£100.
Samaritan Free Hospital, Out-Patient Surgeon.
Royal London Ophthalmic Hospital, Clinical Assistants.
Royal London Ophthalmic Hospital, Assistant Surgeon.
North-West London Hospital, Assistant Surgeon.

Colonel Lyon-Campbell, R.E., has been appointed house governor of the Royal National Hospital for Consumption, Ventnor. There were a great many candidates, the contest was a keen one, and we congratulate Colonel Lyon-Campbell on his election. He will find much good work to do at Ventnor, and we hope that before he takes office he will make a tour of some of the principal hospitals, and then vigorously set himself to work to raise the institutions in the Isle of Wight to the highest pitch of efficiency.

The latest workhouse scandal comes from Christchurch; it concerned the death of an old man aged 60, who was allowed to lie, ill and moaning, without proper food and attention, from Saturday night till Monday morning. The man died, and an inquest was held. The jury concluded that the state of things in the workhouse requires investigation. This is a very bad case of workhouse neglect, and speaks volumes for the need of the guardians being of a better class, and the unions being more open to outside inspection. Officials and Boards are too much inclined to shut out the public, yet surely the ratepayers have as much right to know that their money is properly spent, as have the subscribers to voluntary charities. The system in small towns is often very bad, and too much power is placed in official hands.

The Birmingham Committee on Hospital Reform sat again on the 15th, and examined Mr. Henry Eales, M.R.C.S., of the Birmingham Eye Hospital. In the course of his remarks Mr. Eales said that the patients were questioned by the assistant secretary, who, if doubtful as to their eligibility, referred them to the senior secretary. In cases of urgency assistance was given at once, before inquiry. The question of urgency was determined by the surgeon. The standard of eligibility was fixed by the Secretary. Committees liked to boast of the increasing number of cases, but it had been some satisfaction to the medical staff of the Eye Hospital that they had been able to boast during the last year or two of a reduction in the number, and that the hospital was in such a strong position that it could afford to report a decrease. The number refused relief now was very much smaller than it was four years ago. About one a-week, or fifty a-year, were refused. There was not much abuse at the special hospitals. Mr. Gilbert Smith, F.R.C.S., M.D., also gave evidence with regard to the Skin and Lock Hospital. He said they had an excellent lady secretary who made inquiries, and had a wonderful memory for faces. Their system, he thought, was perfect, and there was very little abuse.

